# Cyclophilin B is really a major growth factor in breast milk

**DOI:** 10.1016/j.jbc.2021.101481

**Published:** 2022-01-12

**Authors:** Olivier Delmas

**Affiliations:** Institut National de l’Environnement Industriel et des Risques (INERIS), Verneuil en Halatte, France

Cyclophilin B (CypB) was first described and its sequence was reported by Spik *et al.* in 1991 (1). These authors prepared and characterized recombinant CypB demonstrating that it had prolyl isomerase activity, which was inhibited by cyclosporin (1). At that time, no mitogenic activity was observed for CypB. This may be because of the absence of glycosylation, incorrect folding, denaturation, or to the cellular model used in these initial exploratory experiments (3T3). We wish to report that an earlier unpublished study (2) showed that CypB has growth factor activity on CCL-39 cells at around 30 ng/ml, similar to EGF, acidic FGF, and basic FGF. The mitogenic activity of CypB was found to be inhibited by Cyclosporin A (CsA), which binds to CypB at about 0.8 mol per mol. Inhibition was complete when CsA was added at a 70-fold excess (FGF activity was not inhibited by CsA). From the data obtained, the initial concentration of CypB in milk is at least 25 ng/ml (EGF concentration has been reported in the range 24–37 ng/ml). CypB is therefore one of the major growth factors in breast milk, sensitive to CsA. More recently published reports indicate a role for cyclophilin A and CypB in cell growth and signal transduction (3, 4). But direct mitogenic activity has never been published. It may act through isomerization of a proline residue of p38MAPK (5). CypB may play roles during lactation, innate development, or necrotizing enterocolitis.

## Conflict of interest

The author declares no conflicts of interests with the contents of this article.
